# Targeted therapy for LIMD1-deficient non-small cell lung cancer subtypes

**DOI:** 10.1038/s41419-021-04355-7

**Published:** 2021-11-11

**Authors:** Kathryn Davidson, Paul Grevitt, Maria F. Contreras-Gerenas, Katherine S. Bridge, Miguel Hermida, Kunal M. Shah, Faraz K. Mardakheh, Mark Stubbs, Rosemary Burke, Pedro Casado, Pedro R. Cutillas, Sarah A. Martin, Tyson V. Sharp

**Affiliations:** 1 Barts Cancer Institute, Queen Mary University of London, John Vane Science Centre, Charterhouse Square, London, EC1M6 BQ UK; 2 York Biomedical Research Institute, University of York, Wentworth Way, York, YO10 5DD UK; 3 Department of Bioengineering, Imperial College, London, UK; 4 Cancer Research UK Cancer Therapeutics Unit, The Institute of Cancer Research, 15 Cotswold Road, Sutton, SM2 5NG UK

**Keywords:** Targeted therapies, Non-small-cell lung cancer

## Abstract

An early event in lung oncogenesis is loss of the tumour suppressor gene *LIMD1* (LIM domains containing 1); this encodes a scaffold protein, which suppresses tumorigenesis via a number of different mechanisms. Approximately 45% of non-small cell lung cancers (NSCLC) are deficient in LIMD1, yet this subtype of NSCLC has been overlooked in preclinical and clinical investigations. Defining therapeutic targets in these LIMD1 loss-of-function patients is difficult due to a lack of ‘druggable’ targets, thus alternative approaches are required. To this end, we performed the first drug repurposing screen to identify compounds that confer synthetic lethality with LIMD1 loss in NSCLC cells. PF-477736 was shown to selectively target LIMD1-deficient cells in vitro through inhibition of multiple kinases, inducing cell death via apoptosis. Furthermore, PF-477736 was effective in treating LIMD1^−/−^ tumours in subcutaneous xenograft models, with no significant effect in LIMD1^+/+^ cells. We have identified a novel drug tool with significant preclinical characterisation that serves as an excellent candidate to explore and define LIMD1-deficient cancers as a new therapeutic subgroup of critical unmet need.

## Introduction

Lung cancer remains the most common cancer in the Western world with ~2 million cases reported worldwide each year [1]. The most frequent type of lung cancer is non-small cell lung cancer (NSCLC), accounting for 84% of total cases, the majority of which are either lung adenocarcinomas (LUAD) or lung squamous cell carcinomas (LUSC) [1, 2]. The 5-year survival of lung cancer patients is only 19%, with minimal improvement in the past 30 years. Recent breakthroughs in immunotherapies and immune checkpoint blockade for lung and several other cancers is encouraging [3–5]. Furthermore, advances in targeted therapy has led to the advent of highly specific targeted treatments, such as tyrosine kinase inhibitors [6]. When used in combination with immunotherapy, this has achieved significant survival benefit for select patient subgroups [7]. However, only a proportion of patients will benefit (~10–40% depending on cancer type), with the overall survival rates remaining largely unchanged [8–12]. This highlights the clear need for novel biomarkers and improved targeted therapies that effect wider patient populations beyond those aided by current approaches [13]. Furthermore, the plethora of mechanisms underlying lung cancer development and progression still remain largely unknown. Driver alterations have not yet been defined in ~40% of lung cancers. Although mutations in several well-known oncogenes and tumour suppressor genes have been detected in certain lung cancers, a large proportion of patients do not contain these common truncal mutations [14–17]. An improved understanding of lung cancer drivers and enhanced treatment options are urgently needed.

*LIMD1* (LIM domains containing 1) is a tumour suppressor gene encoded at the 3p.21.3 genomic locus, which is frequently ablated early in lung cancer development. Reduced *LIMD1* copy number alterations in LUAD correlate with poor patient prognosis [18]. Furthermore, *LIMD1*^*−/−*^ mice develop increased numbers and larger volumes of lung adenomas following exposure to the carcinogen urethane or upon crossing with *KRAS*^*G12D*^ mice, highlighting loss of *LIMD1* as a major driver in lung cancer and potential LUAD and LUSC susceptibility gene [18, 19]. Human lung cancers deficient in *LIMD1* expression represent 50% and 85% of LUAD and LUSC, respectively [18], and have been completely overlooked in preclinical studies. This biomarker signifies a new and exciting avenue for investigation in lung cancer biology and importantly a novel treatment strategy for LIMD1-deficient tumours.

LIMD1 is a member of the Zyxin family of LIM-domain proteins, which feature three tandem LIM domains at the C-terminus that facilitates protein-protein interactions and an unstructured N-terminal pre-LIM region [20]. Whilst LIMD1 has no enzymatic function, it plays an important role in modulating many essential homoeostatic processes by operating as a molecular scaffold [18, 21–29]. We have shown a critical role of LIMD1 as a core component of the microRNA-induced silencing complex (miRISC) [24], and in regulating the hypoxic response by mediating efficient degradation of HIF-1α through simultaneous binding of HIF prolyl-hydroxylases and the Von-Hippel Lindau protein (pVHL) [23, 30]. In addition, LIMD1 binds to and enhances the function of the retinoblastoma protein (pRB), thereby acting as a corepressor blocking E2F1-driven gene transcription and subsequent cell cycle progression [21]. Loss of LIMD1 and its multiple tumour suppressive functions lead to alterations and disruption of these key regulatory pathways, driving cellular transformation and cancer progression.

Despite *LIMD1*’s key homoeostatic functions, the level of ablation in LUAD/LUSC and the large disease burden in lung cancer, there are currently no targeted therapies for LIMD1-deficient cancers. Defining therapeutic targets in these LIMD1 loss-of-function patients is difficult due to no clear ‘druggable’ enzymes that can be targeted, meaning alternative approaches are required. This is further complicated due to the number of diverse pathways impacted upon loss of LIMD1, therefore targeting downstream pathways in isolation is not a feasible option. The concept of synthetic lethality provides a rationale for targeting loss of tumour suppressor genes; whereby cellular vulnerabilities acquired following loss of tumour suppressors are exploited to induce cell death in tumour verses normal tissue. The prime example of this is the use of PARP inhibitors in *BRCA1* mutant cancers, which is now an approved targeted therapy in several cancers [31].

To this end, we have performed the first proof-of-concept drug repurposing screen to identify synthetically lethal compounds with LIMD1 loss. Drug repurposing is an attractive option as a significant amount of preclinical data and safety profiling have already been generated for these compounds, allowing expedited clinical trials for alternative indications [32].

From our compound library screen, we identified a multi-kinase inhibitor, PF-477736 that selectively kills LIMD1-negative cells compared to LIMD1-proficient cells. Whilst this inhibitor was designed as a checkpoint kinase 1 (Chk1) inhibitor, we have shown that Chk1 inhibition does not confer the synthetic lethal interaction with LIMD1 loss in these cells. Instead, our data indicate that this inhibitor affects a broad spectrum of kinases, inducing significant changes to the phosphoproteome specifically in LIMD1-negative cells. Finally, we show that this inhibitor has therapeutic potential in LUAD. This study provides proof-of-concept that LIMD1 expression can be used as a stratification marker for treatment, identifying a large group of lung cancer patients that could benefit from a targeted therapy against *LIMD1* loss.

## Results

### Drug repurposing screen identified PF-477736 as selective inhibitor of LIMD1^−/−^ cells

**Fig. 1:**
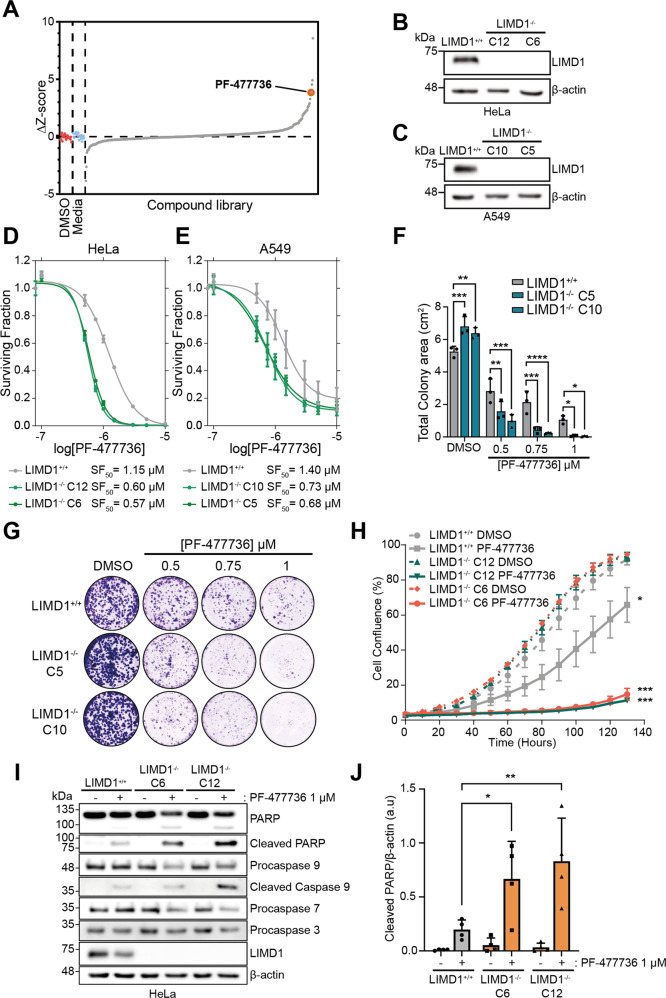
PF-477736 is a selective inhibitor of *LIMD1-*deficient cells. **A** Waterfall plot of ∆*Z*-Scores from compound library screen. Isogenic LIMD1^−/−^ and control lines were treated with a 1 µM dose and cell viability measured after 4 days (*n* = 3). **B**, **C** Immunoblot of CRISPR-Cas9-generated LIMD1^−/−^ HeLa and A549 cell lines. **D**, **E** Dose–response curves of PF-477736 in A549 and HeLa isogenic LIMD1^−/−^ lines. Cells were drugged twice over 4 days before measuring cell viability and calculating surviving fraction (*n* = 3). **F**, **G** Colony formation assay of A549 isogenic LIMD1^−/−^ cells following treatment of PF-477736 for 10 days. Cells were treated every 2 days with indicated concentration of PF-477736 before fixation and staining (*n* = 3, two-way ANOVA). **H** Growth of HeLa isogenic LIMD1^−/−^ measured using Incucyte Zoom following 1 µM treatment with PF-477736 (*n* = 3; one-way ANOVA comparing AUC for each curve). **I** Western blot of PARP and caspase cleavage in HeLa isogenic LIMD1^−/−^ lines treated with PF-477736 for 48 h (*n* = 4). **J** Quantification of western blot of apoptosis markers in HeLa isogenic LIMD1^−/−^ lines treated with PF-477736 for 48 h (*n* = 4, two-way ANOVA). ns *p* > 0.05, *p ≤ 0.05, **p ≤ 0.01, ***p ≤ 0.001, ****p ≤ 0.0001.

To identify compounds that selectively target LIMD1^−/−^ cells, we screened CRISPR-Cas9-generated isogenic LIMD1^+/+^ and LIMD1^−/−^ HeLa cells with a compound library of 485 small molecules. This drug library was collated to include FDA-approved drugs, clinical candidates and compounds against known cancer pathways [33, 34]. Cells were treated with a 1 μM concentration of each compound, and cell viability was determined after 4 days. Upon determination of the ∆*Z*-scores we identified the Chk1 inhibitor PF-477736 as our lead hit, as it had one of the highest ∆*Z*-scores, causing significantly decreased cell viability in LIMD1^−/−^ cells, compared to the LIMD1^+/+^ cells, as well as not showing overt toxicity in the LIMD1^+/+^ line (Fig. 1A). We validated this synthetic lethal interaction across a range of concentrations, in multiple LIMD1^−/−^ cell clones (Fig. 1B–E). Of note, this phenotype was validated in our isogenic pair of CRISPR-Cas9-generated LUAD A549 LIMD1^+/+^ and LIMD1^−/−^ cells (Fig. 1E) indicating this effect was not cell line specific and was relevant in the context of lung cancer biology. In both LIMD1 isogenic cell models, there was a ~2-fold selectivity towards LIMD1^−/−^ cells compared to LIMD1^+/+^ controls (Fig. 1D, E) demonstrated by a significant difference in SF_50_ values (Fig. S1A, B). In addition, we validated this effect in the clear-cell renal cell carcinoma cell line RCC48, using RNAi depletion of *LIMD1*. Here we observed increased sensitivity of these sh*LIMD1* cells to PF-477736 treatment, which could be rescued upon the expression of an RNAi-resistant Flag-His-tagged LIMD1 indicating that this increased sensitivity is specific to LIMD1 loss (Fig. S1C). We further validated this effect using long-term clonogenic drug assays in A549 and HeLa cells, which showed a significant decrease in clonogenic potential for LIMD1^−/−^ cells compared to the LIMD1^+/+^ upon PF-477736 treatment (Figs. 1F, G and S1D, E). In addition, we treated these isogenic lines with 1 μM PF4 and measured cell proliferation using bright field imaging on Incucyte Zoom; whilst there was a modest reduction in proliferation in the LIMD1^+/+^ lines upon PF-477736 treatment (~1.6-fold reduction in AUC), this effect was significantly enhanced in both LIMD1^−/−^ clones (~7-fold reduction in AUC) (Fig. 1H). Notably, PF-477736 treatment induced ‘blebbing-like’ structures of cell membranes in LIMD1^−/−^ cells, suggesting that cells may be undergoing increased apoptosis (Fig. S1F). This was confirmed by increased PARP cleavage and caspase activation in LIMD1^−/−^ clones compared to LIMD1^+/+^ controls upon PF-477736 treatment (Figs. 1I, J and S1G–J). Annexin V staining identified the increased early and late apoptotic cell populations in PF-477736 treated LIMD1^−/−^ cells **(**Fig. S1K**)**. Taken together, these data indicate that PF-477736 treatment can selectively target LIMD1^−/−^ and RNAi-driven deficient cells; identifying apoptosis as the mechanism of cell death.

### PF-477736 selectively kills LIMD1^−/−^ cells independent of Chk1 inhibition

**Fig. 2:**
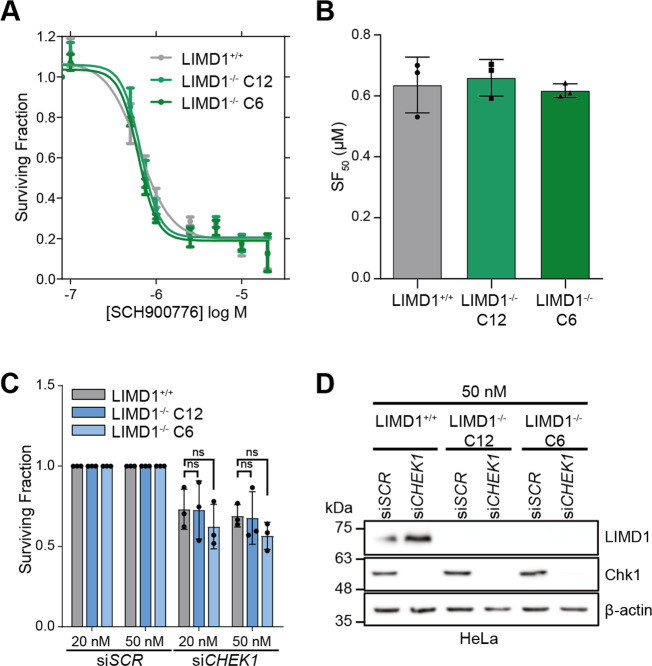
PF-477736 selectively kills *LIMD1*^*−/−*^ cells independent of Chk1 inhibition. **A** Dose–response curve of SCH900776 in HeLa isogenic LIMD1^−/−^ lines. Cells were treated for 4 days before measuring viability and calculating surviving fraction (*n* = 3). **B** Bar chart of SF_50_ values from panel (**A**) (*n* = 3, one-way ANOVA). **C** Surviving fraction of HeLa isogenic LIMD1^−/−^ lines transfected with siRNA against *CHEK1* at 50 nM and 20 nM for 72 h (*n* = 3, two-way ANOVA). ns *p* > 0.05. **D** Immunoblot of Chk1 and LIMD1 in HeLa isogenic LIMD1^−/−^ lines transfected with siRNA against *CHEK1* (20 nM, 72 h) (*n* = 3).

PF-477736 was originally developed for use in combination therapy with DNA damage-inducing agents as a sub-nanomolar ATP-competitive inhibitor of Chk1 (*K*_*i*_ = 0.49 nM) [35]. However, neither of the two Chk1 inhibitors in the compound library (AZD7762 and LY2603618) were identified as hits in the screen (Fig. S2A). We reasoned this may have been an artefact of the single-concentration dose used in the primary screen. We tested an alternative Chk1 inhibitor, SCH900776 (MK-8776, Chk1 *K*_*i*_ = 3 nM), to establish if this caused LIMD1^−/−^-specific cell death (Fig. 2A, B). We did not observe any difference in cell viability upon SCH900776 treatment between our HeLa LIMD1 isogenic lines. Transient RNAi knockdown of *CHEK1* in our isogenic lines was also performed, and once again did not induce any differences in cell viability between these lines (Fig. 2C, D). These data indicate that Chk1 inhibition is not synthetically lethal with LIMD1 loss, and therefore the effects we see with PF-477736 are likely to be through off-target inhibition of an alternative kinase or kinases.

### PF-477736 is a broad-spectrum kinase inhibitor that elicits LIMD1^−/−^-specific cellular changes in the phosphoproteome

**Fig. 3:**
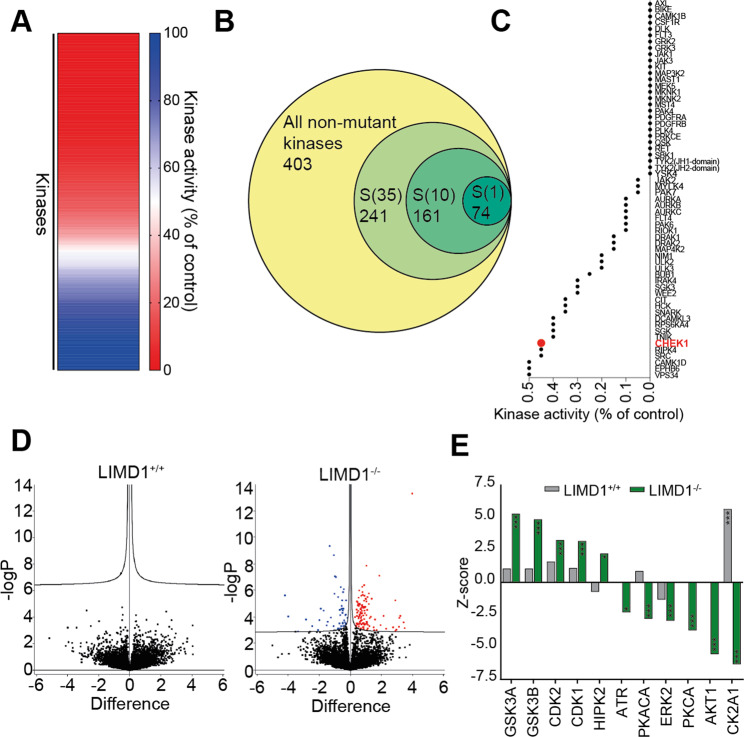
PF-477736 is a broad-spectrum kinase inhibitor that elicits *LIMD1*^*−/−*^-specific cellular changes in the phosphoproteome. **A** Heatmap showing remaining kinase activity of a panel of in vitro kinases upon 3 µM PF4-77736 treatment in DiscoverX KINOMEscan assay. **B** Venn diagram representing the proportion and number of kinases inhibited to less than 35%, 10% and 1% of control activity. **C** Waterfall plot of kinases most inhibited by PF-477736 (3 µM) in the in vitro kinase assay. **D** Volcano plot of phosphosite changes between 1 µM PF-477736-treated and DMSO control lysates in isogenic HeLa lines. Cells were harvested following 1 h drug treatment. No significant phosphosite changes were induced by PF-477736 in the LIMD1^+/+^ cell line, compared with 54 reduced and 119 increased phosphosites in the LIMD1^−/−^ cell line. Cut-off point for statistically significant phosphosite is a false discovery rate of >0.05 and S0 of 0.01 (*n* = 3). **E** Kinase substrate enrichment analysis (KSEA) of phosphoproteomics shows kinases significantly affected by PF-477736 treatment in LIMD1^+/+^ (grey) or LIMD1^−/−^ cells (green) *p ≤ 0.05, **p ≤ 0.01, ***p ≤ 0.001.

With the aim of identifying the kinase inhibited by PF-477736 that is responsible for the observed synthetic lethality with LIMD1 loss, we tested the activity of PF-477736 on a panel of 403 recombinant non-mutant kinases and 59 clinically relevant disease-mutant kinases using the DiscoverX KINOMEscan platform, which involves an in vitro ATP-independent competition assay to measure kinase activity [36]. Optimum inhibitor concentration is defined as 3–10-fold higher than the *K*_*i*_ of targeted interactions, therefore as *in cellulo* we observe the strongest synthetic lethal interaction at 1 μM, we opted to perform profiling at a concentration of 3 μM. Surprisingly, 303 out of the 468 kinases tested were inhibited by over 50%, upon PF-477736 treatment compared to the DMSO control (Fig. 3A). We observed a wide range of inhibition across the kinases tested, with 18.4% of kinases inhibited by 99%, further highlighting the broad specificity of this inhibitor (Fig. 3B). A percentage of control value of <1% indicates a *K*_*d*_ value of <30 nM. Chk1 activity was reduced to 0.45% by PF-477736, and a number of other kinases were more potently inhibited than Chk1 (Fig. 3C). Next, in an endeavour to elucidate which kinases are inhibited by PF-477736 in the cell as opposed to the in vitro study, we analysed the phosphoproteome in our isogenic lines upon drug treatment. Following 1 h treatment with PF-477736 we did not observe any significant changes in the phosphoproteome of HeLa LIMD1^+/+^ cells; strikingly however, there were numerous significant changes occurring in the HeLa LIMD1^−/−^ cells (Fig. 3D). Principal component analysis (PCA) on these data showed no separation between treated and untreated samples in the LIMD1^+/+^ cell line, but a clear separation in the LIMD1^−/−^ cell line (Fig. S3A, B). This result emphasises that PF-477736 treatment elicits significant phosphorylation changes specifically upon loss of LIMD1. We next utilised kinase-substrate enrichment analysis (KSEA) to infer kinase activities from our quantitative phosphoproteomics data [37]. This analysis identified a number of kinases (CK2A1, CDK1, PKCA, ERK1 and Akt1) that were significantly more active in LIMD1^−/−^ cells compared to LIMD1^+/+^ controls (Fig. S3C). Furthermore, CK2A1, PKCA and Akt1 were all significantly inhibited following PF-477736 treatment specifically in LIMD1^+/+^ cells. Interestingly, LIMD1^+/+^ cells exhibited increased CK2A1 activity after treatment (Fig. 3E). In our DiscoverX KINOMEscan data, these kinases were inhibited by 90.1%, CK2A1; 89%, PKCA and 77%, Akt1 (Fig. 3A–C). To determine whether loss of these kinases could induce synthetic lethality upon LIMD1 loss, we knocked down the expression (via siRNA) of these kinases in combination, however, there were no significant changes in cell viability between our isogenic lines with each of these knock-downs (Fig. S3D). We next reasoned that knocking down the kinase levels significantly with siRNA could possibly still leave very low levels of protein and activity that could still maintain viability, and thus opted to examine small-molecule inhibitors against the targets (both individually and in combination at a dose that corresponds with SF_80_). This drug-targeted approach was based on inhibitors: MK-2206, AKT inhibitor; Silmitasertib, a casein kinase 2 inhibitor; and Gouml 6983 (Go-6983), a broad-spectrum PKC inhibitor. These drug combinations did not show any selectivity towards LIMD1-deficient cells, indicating that other pathways are involved (Fig. S3E, F). Regardless of not identifying a specific kinase or pathway inhibited by PF-477736 in LIMD1-deficient cells, these data do indicate that loss of LIMD1 increases the activity of a broad panel of kinases including CK2A1, PKCA and Akt1, which were collectively inhibited by PF-477736 treatment leading to increased apoptosis in LIMD1^−/−^ cells.

### PF-477736 treatment as an initial proof-of-concept inhibitor of LIMD1-deficient lung cancers

**Fig. 4:**
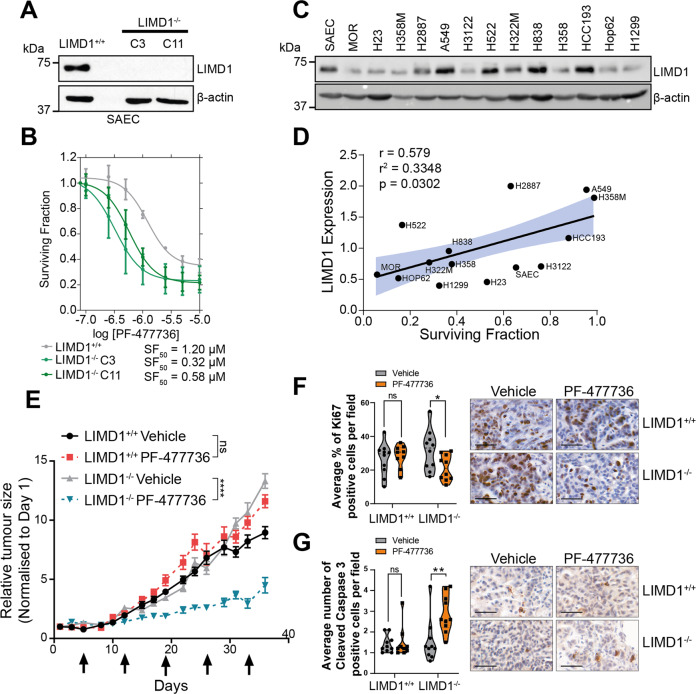
PF-477736 treatment is proof-of-concept inhibitor of *LIMD1-*deficient lung cancers. **A** Immunoblot of isogenic LIMD1^−/−^ small airway epithelial cells (SAEC) and control. **B** Dose–response of PF-477736 in isogenic LIMD1^−/−^ SAEC. Cells were treated twice prior to measuring cell viability and calculating surviving fraction (*n* = 3). **C** Immunoblot of LIMD1 in a panel of lung adenocarcinoma cell lines (representative blot from *n* = 3). **D** Pearson’s correlation coefficient between LIMD1 protein expression and surviving fraction of indicated cell line after treatment with 1 µM PF-477736. **E** Relative tumour growth of subcutaneous A549 isogenic xenografts implanted into the flank of NOD/SCID mice. Mice were treated twice on indicated days with vehicle or PF-477736 (7.5 mg/kg per dose) (*n* = 10 per group). **F**, **G** Immunohistochemical staining and scoring of Ki67 (**F**) and cleaved caspase-3 (**G**) in mouse xenograft tumours (*n* = 10 per group, two-way ANOVA).

Previous work from our group has extensively characterised the role of LIMD1 as a tumour suppressor in lung cancer, therefore we investigated the potential of PF-477736 as a therapeutic option against LIMD1-deficient lung cancer cell lines. We have already shown selectivity of PF-477736 against LIMD1^−/−^ A549 cells, a NSCLC adenocarcinoma cell line (Fig. 1E). To test PF-477736 in non-transformed lung cells, we generated LIMD1^−/−^ small airway epithelial cells (SAEC) that had been immortalised by overexpressing Bmi1 (SAEC-Bmi1). These SAEC are a mixture of both type I and type II alveolar cells, thereby serving as an appropriate model of LUAD progenitor cells, which have been CRISPR-Cas9 edited to express a N-terminal truncated form of LIMD1 in significantly lower levels compared to non-targeting controls, thereby representing an in vitro model of LIMD1 loss in the development of adenocarcinoma (Fig. 4A). Comparable with our other isogenic cell lines, we observed ~2–4-fold selectivity for SAEC LIMD1^−/−^ cells (Fig. 4B). Next, we treated a panel of LUAD cell lines exhibiting a range of LIMD1 protein expression levels, with PF-477736 at 1 μM (Fig. 4C, D). We observed a significant positive correlation (Pearson’s correlation coefficient = 0.579, *p* = 0.0302) with LIMD1 protein expression and surviving fraction, thereby indicating that it may be possible to use LIMD1 expression as a biomarker to stratify patients for targeted therapy treatment efficacy (Fig. 4D). To test the efficacy of PF-477736 in vivo, we inoculated NOD/SCID mice with our A549 isogenic lines subcutaneously and treated with PF-477736 twice on indicated days. LIMD1^+/+^ tumours were unaffected by PF-477736 treatment in vivo, however we observed a significant decrease in tumour growth in the LIMD1^−/−^ tumours upon treatment (Figs. 4E and S4A, B). Staining of these tumours with markers for cell proliferation (Ki67, Figs. 4F and S4C) and apoptosis (cleaved caspase-3, Figs. 4G and S4D), revealed that PF-477736 selectivity inhibits proliferation in LIMD1-deficient lung xenografts and increases apoptosis within these tumours, in agreement with our in vitro data.

To summarise our results, we have identified that PF-477736 selectively induces apoptosis in LIMD1^−/−^ cells by targeting multiple susceptibility pathways, whilst sparing LIMD1^+/+^ cells. This is the first evidence supporting a targeted therapeutic approach for the treatment of lung cancers with reduced or loss of LIMD1 expression.

## Discussion

Lung cancer has a staggering disease burden, with a clear need for targeted therapies that are effective in wider patient populations. Treating/targeting the loss of the tumour suppressor gene *LIMD1* is an attractive therapeutic option as we have identified copy number alterations in ~47% of LUAD patients [18]. Here we show a proof-of-concept study identifying PF-477736 as a selective inhibitor of LIMD1-deficient cells (Fig. 1). Crucially, we observe a correlation with LIMD1 expression and drug sensitivity, indicating that we may be able to use LIMD1 expression as a biomarker for new targeted therapies, and thus indicate potential treatment response.

PF-477736 is a Chk1 inhibitor, however our results indicate a large number of kinases are potently inhibited in vitro and *in cellulo* by PF-477736 (Fig. 3). This finding is perhaps not surprising, as an expanding number of studies have shown that several ATP-pocket kinase inhibitors that have been used in clinical trials exhibit off-target mechanisms of action distinct from the primary drug target [38]. Our data suggest that targeting multiple pathways is required to target LIMD1^−/−^ cells; previous work from our group and others have highlighted a number of pathways that LIMD1 plays a critical role in regulating [24, 39]. Therefore, it is unsurprising that targeting just one LIMD1-regulated pathway is insufficient to recapitulate PF-477736-induced cell death. Broad-spectrum inhibitors have been successfully utilised in other cancers, such as dual inhibition of PDGFRa and FGFR2 by pazopanib in SMARCB1-deficient rhabdoid tumours [40, 41]. This fits within our model of LIMD1 being a nodal gene, loss of which re-orchestrates numerous homoeostatic cellular pathways, which requires inhibition of multiple deregulated pathways to induce cell death. For our analysis we focused on protein kinases as PF-477736, which was designed as an inhibitor of the ATP moiety to block kinase function, however, there remains the possibility that PF-477736 is acting on a non-kinase enzyme or a kinase not covered by our analysis.

This proof-of-concept study has identified, for the first time, a selective inhibitor of LIMD1-deficient cells. We have shown that it is possible to target these cells with a small molecule, allowing for the potential targeted treatment of a large proportion of lung cancer patients with LIMD1-deficient tumours. Targeting such cancers is particularly attractive as LIMD1 loss has been further observed in breast, cervical, gastric, renal, and head and neck cancers [42–47]. Our study has identified a novel therapeutic strategy based on LIMD1 status, which exploits the loss of this tumour suppressor, offering the potential for targeted treatment for this large cancer-patient population and significantly reduce disease burden worldwide.

## Materials and methods

### Cell culture

Cells were maintained in DMEM (HeLa, A549) or RPMI (HCC193, H1299, Hop62, H358, H838, H358M, H2887, H522, H322M, H23, MOR, H3122) (Sigma) supplemented with 10% FCS and 1% penicillin/streptomycin solution in a humidified 37 °C incubator with 5% CO_2_. SAEC were maintained in complete Airway Epithelial Cell Medium (ATCC). SAEC cells were immortalised using pFLRu-Bmi-1. Parental stocks of each line were obtained from ATCC. All cell cultures were regularly tested for mycoplasma. Isogenic lines were authenticated by STR profiling post editing.

### Drug screen

Cells were plated in 96-well plates at an optimised cell density, and after 24 h, treated with vehicle (0.01% DMSO) or the compound library at a final concentration of 1 μM. Cells were dosed again after 48 h. Cell viability was assessed using CellTitre-Glo (Promega) after 4 days of drug exposure, according to manufacturer’s instructions. Luminescence readings from each well were log transformed and normalised according to the median signal on each plate and then standardised by *Z*-score statistic, using the median absolute deviation to estimate the variation in each screen. *Z*-scores were compared to identify compounds that cause selective loss of viability in LIMD1^−/−^ cells compared to LIMD1^+/+^ cells.

### Drug validation experiments

PF-477736 was provided by Pfizer as part of Pfizer Global Medicine Grant 60411717. SCH900776, MK-2206 Go-6983, Silmitasertib were purchased from Selleckchem. In line with the drug-screen protocol, cells were seeded into a 96-well plate and treated 24 h later with a concentration range of drug, or DMSO control. Cells were re-dosed with the drug after 48 h and cell viability was determined after 4 days of drug exposure using CellTitre-Glo. For combination studies, SF_80_ was calculated using the formula SF_80_ = (80/(100 − 80)^√Hill slope^) × SF_50_. Cells were plated and dosed as above before measuring viability with CellTitre-Glo.

### Colony formation assay

Cells were seeded at 1000 cells per well in 6-well plates. At 24 h post seeding, cells were treated with drug or vehicle control. Drug-containing media was refreshed every 2 days and cells were fixed in methanol after 10 days of treatment. Colonies were stained with 0.05% crystal violet and counted manually or total colony area was calculated using ImageJ.

### Incucyte

Growth curves were generated using the Incucyte ZOOM live-cell imaging platform (Essen Bioscience). Cells were seeded into 96-well plates and were drugged 24 h later with either 1 μM PF-477736 or DMSO control. Images were captured at 10x magnification on the Incucyte every 2 h. Drug was refreshed after 68 h. Cell confluence per well was calculated using the Incucyte ZOOM software (Essen Bioscience).

### Protein analysis

Cell pellets were lysed in RIPA buffer (150 nM NaCl, 1% (v/v) IGEPAL, 0.5% (w/v) deoxycholic acid, 0.1% (w/v) SDS, 50 mM Tris) supplemented with protease and phosphatase inhibitors. Protein was quantified using the Pierce BCA protein assay kit (Thermo Fisher Scientific). Lysates were electrophoresed on acrylamide gels of appropriate acrylamide percentage, transferred onto PVDF membranes and immunoblotted using the following antibodies: LIMD1 (in house), β-actin (Sigma #A1978), Total PARP (CST #9532), Cleaved PARP (CST #6704), Caspase 7 (CST #12827), Caspase 9 (CST #9508), Caspase 3 (CST #9668) and Cleaved Caspase 9 (CST #7237), Chk1 (CST #2360). Anti-IgG horseradish peroxidase (Dako) and chemiluminescent detection (Thermo Fisher Scientific) were used to develop immunoblots.

### siRNA transfections

siRNA targeting Chk1, CSNK2A1, AKT1, PKCA, PLK1 and non-targeting control were obtained as SMARTPools from Dharmacon. siRNAs were transfected using Lipofectamine RNAiMax (Invitrogen) according to manufacturer’s instructions. Then, 96-well plates were used for cell viability endpoints, and 12-well or 6-well plates were used for protein extraction to determine the protein knockdown by immunoblot.

### Annexin V staining

Cells were seeded into 10 cm dishes at a density of 1 × 10^4^ cells/dish; 24 h later, dishes were treated with 1 μM PF-477736 or DMSO vehicle control. After 48 h, cells were trypsinised, harvested (including those in media and PBS washes), counted and 1 × 10^6^ cells were resuspended into 1x annexin-binding buffer (Thermo Fisher Scientific). Then, 2 μL Alexa Flour 488 Annexin V (Thermo Fisher Scientific) and 1 μL 100 μg/mL PI were added to each 100 μL of cell suspension and incubated for 15 min. Single-stained and unstained controls were stained accordingly. Samples were run through the BD LSR Fortessa flow cytometer (Becton Dickinson, USA), recording 10,000 events for each sample. Data were analysed using FlowJo version 10 (FlowJo LLC).

### Kinase profiling

Kinase profiling was conducted by DiscoverX (USA). PF-477736 was dissolved in DMSO and diluted to 3 mM (1000x screening concentration) for shipment. Profiling was performed by Discover X as per their in-house protocols.

### Phosphoproteomics

Mass spectrometry analysis was conducted as described by Casado et al. (2018) [48]. Briefly, HeLa LIMD1^+/+^ and LIMD1^−/−^ were seeded into 10 cm dishes at 7 × 10^5^ cells/dish. At 48 h post seeding, dishes were treated with either 1 μM PF-477736 or DMSO vehicle control for 1 h. Cells were washed 3x in ice-cold PBS containing protease and phosphatase inhibitors (1 mM NaF and 1 mM Na_3_VO_4_). Dishes were lysed in 200 μL of lysis buffer (8 M urea in 20 mM HEPES (pH 8.0) supplemented with 1 mM Na_3_VO_4_, 1 mM NaF, 1 mM β-glycerol phosphate and 2.5 mM Na_2_H_2_P_2_O_7_) and cells were scraped and transferred into Protein LoBind tubes (Eppendorf). Samples were sonicated for 10 cycles (30 s on and 40 s off) in a Diagenode Bioruptor^®^ Plus. Samples were centrifuged at 20,000*g* for 10 m, 4 °C and supernatant was transferred to 1.5 mL Protein LoBind tubes. BCA assay was conducted to quantify protein concentration.

Protein suspensions of 400 µg of protein in a volume of 200 µL were subjected to cysteine reduction and alkylation using sequential incubation with 10 mM dithiothreitol (DDT) and 16.6 mM iodoacetamide (IAM) for 1 h and 30 min, respectively, at 25 °C with agitation. The urea concentration in the protein suspensions was reduced to 2 M by the addition of 600 µL of 20 mM HEPES (pH 8.0), and 100 μL of equilibrated trypsin beads were added and samples were incubated overnight at 37 °C. Trypsin beads (50% slurry of TLCK-trypsin) were equilibrated with 3 washes with 20 mM HEPES (pH 8.0). The following day, trypsin beads were removed by centrifugation (2000*g* at 5 °C for 5 min) and samples were desalted using Oasis HLB cartridges (Waters).

Briefly, cartridges set in a vacuum manifold device, with a pressure adjusted to 5 mmHg, were conditioned with 1 mL acetonitrile (ACN) and equilibrated with 1.5 mL of wash solution (0.1% trifluoroacetic acid (TFA), 2% ACN). Then, peptide solutions were loaded into the cartridges and washed twice with 1 mL of wash solution. Peptides were eluted with 0.5 mL of glycolic acid buffer A (1 M glycolic acid, 5% TFA, 50% ACN).

For phosphopeptide enrichment, eluents were normalised to 1 mL with glycolic acid buffer B (1 M glycolic acid, 5% TFA, 80% ACN) and incubated with 25 µL of TiO_2_ solution (500 mg TiO_2_ beads in 500 µL of 1% TFA) for 5 min at room temperature (RT). TiO_2_ beads were packed by centrifugation into empty spin columns previously washed with ACN. TiO_2_-bead pellets were sequentially washed by centrifugation (1500*g* for 3 min) with 100 µL of glycolic acid buffer B, ammonium acetate buffer (100 mM ammonium acetate in 25% ACN) and twice with neutral solution (10% ACN). Spin tips were transferred to fresh tubes, and phosphopeptides were eluted by adding 50 µL of elution solution (5% NH_4_OH, 7.5% ACN) and centrifuging the spin tips at 1500*g* for 3 min. This elution step was repeated a total of four times. Finally, samples were frozen in dry ice for 15 min, dried in a SpeedVac vacuum concentrator and stored at −80 °C.

Peptide pellets were reconstituted in 13 µL of reconstitution buffer (20 fmol/µL enolase in 3% ACN, 0.1% TFA) and 5 µL were loaded twice onto an LC-MS/MS system consisting of a Dionex UltiMate 3000 RSLC coupled to Q Exactive™ Plus Orbitrap Mass Spectrometer (Thermo Fisher Scientific) through an EASY-Spray source. Chromatographic separation of the peptides was performed using the mobile phases A (3% ACN; 0.1% FA) and B (99.9% ACN; 0.1% FA). Peptides were loaded in a μ-pre-column and separated in an analytical column using a gradient running from 3% to 23% B over 60 min. The UPLC system delivered a flow of 2 µL/min (loading) and 250 nL/min (gradient elution). The Q Exactive Plus operated a duty cycle of 2.1 s. Thus, it acquired full scan survey spectra (*m*/*z* 375–1500) with a 70,000 FWHM resolution followed by data-dependent acquisition in which the 15 most intense ions were selected for HCD (higher-energy collisional dissociation) and MS/MS scanning (200–2000 *m*/*z*) with a resolution of 17,500 FWHM. A dynamic exclusion period of 30 s was enabled with a *m*/*z* window of ±10 ppm.

Peptide identification was automated using Mascot Daemon 2.6.0. Thus, Mascot Distiller v2.6.1.0 generated peak list files (MGFs) from RAW data and Mascot search engine (v2.6) matched the MS/MS data stored in the MGFs to peptides using the SwissProt Database (SwissProt_2016Oct.fasta). Searches had a false discovery rate (FDR) of ~1% and allowed 2 trypsin missed cleavages, mass tolerance of ±10 ppm for the MS scans and ±25 mmu for the MS/MS scans, carbamidomethyl Cys as a fixed modification and PyroGlu on N-terminal Gln, oxidation of Met and phosphorylation on Ser, Thr and Tyr as variable modifications.

A label-free procedure based on extracted ion chromatograms (XICs) quantified all identified peptides. Missing data points were minimised by constructing XICs across all LC-MS/MS runs for all the peptides identified in at least one of the LC-MS/MS runs [49]. XIC mass and retention time windows were ±7 ppm and ±2 min, respectively. Quantification of peptides was achieved by measuring the area under the peak of the XICs. Individual peptide intensity values in each sample were normalised to the sum of the intensity values of all the peptides quantified in that sample. Data points not quantified for a particular peptide were given a peptide intensity value equal to the minimum intensity value quantified in the sample divided by 10. Significant differences in phosphopeptide intensities were assessed by student’s *t* test with Benjamini Hochberg multiple testing correction (FDR).

### Kinase-substrate enrichment analysis

Kinase-substrate enrichment analysis (KSEA) was conducted as previously described by Casado et al. (2013) [37]. Briefly, phosphopeptides with a *p* < 0.05 (assessed by *t* test of log_2_-transformed data) were grouped into substrate sets based on the PhosphoSite database. The ‘enrichment’ method was used to infer differences in the abundance of substrate groups across samples. *Z*-score was calculated using the formula (*Z*-score = (*mS* − *mP*)*m^1/2^/δ) where *m* is the size of the substrate group and δ is the standard deviation of the mean abundance across the whole dataset. *Z*-score was converted to a *p* value in Excel.

### Immunohistochemistry and scoring

Formalin-fixed paraffin-embedded (FFPE) mouse tumours were sliced into 4 µm thick sections. Slides were baked overnight at 57 °C prior to dewaxing. Afterwards, slides were put into xylene, 100% ethanol, and in 3% H_2_O_2_/methanol solution for 10 min for endogenous peroxidase inactivation. Antigen retrieval for Ki67 and cleaved caspase-3 was done using a 10 mM citrate buffer (pH 6) for 10 min at high power (700 W) in a microwave. Samples were left at RT for 30 min and blocked in 10% goat serum for 20 min. Primary antibodies (Ki67 Abcam ab16667, Cleaved Caspase-3 CST #9661) were applied at a 1:100 dilution and left incubating for 1 h at RT (Ki67) or overnight at 4 °C (cleaved caspase-3). Anti-rabbit biotinylated secondary antibody (Vector laboratories BA-1000-1.5) was added at a 1:200 dilution for 30 min at RT. Samples were incubated with ABC reagent (Vector laboratories PK-6100) for 20 min at RT. Afterwards slides were developed with DAB solution (Dako K3468) for 5 min. Washes with PBS (2x, 2 min each) were carried out every time slides were incubated with a different reagent.

Finally, samples were counterstained with haematoxylin, differentiated in 1% acid alcohol, dehydrated in 70%, 90% and 100% ethanol, cleared in xylene and mounted using DPX mounting medium. Slides were imaged using the PANNORAMIC 250 Flash III scanner (3D Histech).

For the scoring, ten 50x fields were selected at random so that the tumour regions within the xenograph were uniformly covered. Cleaved caspase-3-stained samples presented a highly apoptotic area at the interphase of the tumour and the stromal regions. Cleaved caspase-3 staining was strong in this area for all samples (regardless of the cell type or the treatment) and was therefore, excluded from the quantifications. For Ki67, the quantification fields were imported into QuPath software and the positive-cell-detection feature was used to detect the total number of cells and the number of DAB-positive and -negative cells. For cleaved caspase-3, ImageJ was used and positive cleaved caspase-3 cells were manually counted using the Cell Counter plugin. Early and late apoptotic cells were counted. Criteria for considering a cleaved caspase-3-positive cell was that the DAB signal should show a defined shape and colocalise with hematoxylin (presence of a nucleus). In some cases, a cell was included where more than one nuclei was observed (late apoptosis).

### Subcutaneous xenograft study

Six-week-old female NOD/SCID mice were purchased from Charles River and housed with food and water ad libitum, five animals were kept in each cage. A549 LIMD1^−/−^ and LIMD1^+/+^ were grown until reaching the beginning of the exponential growth phase and then detached and resuspended in Matrigel 5 mg/mL (Sigma). First, 1 × 10^6^ cells in 100 µL of Matrigel were injected subcutaneously into the mice, 20 with A549 LIMD1^−/−^ and 20 with A549 LIMD^+/+^. Tumour growth was measured three times per week using callipers and the tumour size calculated using the formula *V* = (length^2^ × width)/2. Once the average tumour size was between 150 and 200 mm^3^, each group was randomised into two groups to maintain equal tumour size between groups and once a week they received either PF-477736 (7.5 mg/kg per dose) or vehicle (50 nM sodium acetate and 4% dextrose, pH 4 (Sigma)) both dosed twice a day with 6 h difference. Tumour size and mice weight were monitored three times per week and the experiment was stopped when the tumour size exceeded 1.44 cm^3^. Mice were then culled and the tumours harvested, sectioned longitudinally and each section was fixed in 10% neutral buffered formalin.

### Statistical analysis

Data were normalised to relevant controls as required. Statistical analysis was conducted using GraphPad Prism 8.0, using the appropriate statistical test for the number of groups and type of data generated from the experiment. All the data are shown as mean ± SD of a minimum of three biological replicates. When multiple comparisons have been performed Dunnett’s multiple comparison post hoc tests were performed. Statistical significance is shown using the following nomenclature: ns *p* > 0.05, ^*^p ≤ 0.05, ^**^p ≤ 0.01, ^***^p ≤ 0.001, ^****^p ≤ 0.0001.

## Supplementary information

Supplementary Legends.

Supplemental Fig. 4.

Supplemental Fig. 1.

Supplemental Fig. 2.

Supplemental Fig. 3.

## Data Availability

The mass spectrometry phosphoproteomics and proteomics data generated during this study have been deposited to the ProteomeXchange Consortium via the PRIDE partner repository with the dataset identifier PXD023674. All other data related to this study are available upon request (t.sharp@qmul.ac.uk).

## Materials availability

All materials related to this study are available upon request (t.sharp@qmul.ac.uk).
