# Supplementary material for: Targeted therapy for LIMD1-deficient non-small cell lung cancer subtypes
Source: Cell Death Dis. 2021 Nov 11;12(11):1075. doi: 10.1038/s41419-021-04355-7 (PMC8586256; doi:10.1038/s41419-021-04355-7)
Supplement: Supplementary file 1 — Supplementary Legends. [file 41419_2021_4355_MOESM1_ESM.docx]

***Supplementary Legends***

***Figure S1 PF-477736 is a selective inhibitor of LIMD1 deficient cells.* A-B)** SF_50_ values of PF-477736 in A549 and HeLa isogenic LIMD1^-/-^ lines (n = 3, one-way ANOVA). **C)** SF_50_ values of PF-477736 in shRNA-knockdown and LIMD1-rescue (rrLIMD1) RCC48 cells and representative western blot showing LIMD1 expression. **D-E)** Colony formation assay of HeLa isogenic LIMD1^-/-^ cells following treatment of PF-477736 for 10 days. Cells were treated every 2 days with indicated concentration of PF-477736 before fixation and staining (n = 3, two-way ANOVA). **F)** Bright field images of cells upon 48 hours of PF-477736 treatment (scale bar = 100 µm, representative images from n = 3). **G-J)** Densitometry for the indicated apoptosis markers in HeLa isogenic LIMD1^-/-^ lines treated with PF-477736 for 48 hours. (n = 4, two-way ANOVA). **K)** Bar chart and contour plots of Annexin V/PI staining in HeLa isogenic LIMD1^-/-^ lines. Cells were treated for 48 hours at 1 µM dose of PF-477736 before staining and analysis by flow cytometry (n = 3). ns p>0.05, *p≤0.05, **p≤0.01, ***p≤0.001, **** p<0.0001.

***Figure S2 P—477736 selectively kills LIMD1^-/-^ cells independent of Chk1 inhibition. ­*A)** Alternative Chk1 inhibitors from drug screen in Figure 1A. Surviving fraction determined following 5 days treatment at 1 µM.

***Figure S3 PF-477736 is a broad-spectrum kinase inhibitor that elicits LIMD1^-/-^ specific cellular changes in the phosphoproteome.* A-B)** PCA analysis on phosphoproteome changes shows no separation between treated and untreated samples in LIMD1^+/+^ cells, but a clear separation in LIMD1^-/-^ cells. **C)** Kinase Substrate Enrichment Analysis (KSEA) of phosphoproteomics shows kinases significantly affected between LIMD1^+/+^ and LIMD1^-/-^ with DMSO treatment. **D)** Cell viability of HeLa isogenic lines treated with siRNA against *CSNK2A1*, *AKT1* or *PKCA* at total siRNA concentration of 30 nM (2 siRNAs) or 45 nM (3 siRNAs). Viability was measured 120 hours post transfection and surviving fractions were calculated (n = 3) **E)** Dose response curves of indicated inhibitors in HeLa LIMD1^+/+^ line (n = 2). **F)** Cell viability of HeLa isogenic LIMD1^-/-^ lines treated with inhibitors in combination at their SF_80_ values based upon dose response curves in panel B and PF-477736 at 1 µM as positive control (n = 3, two-way ANOVA). ns p>0.05 *p≤0.05, **p≤0.01, ***p≤0.001

***Figure S4 PF-477736 treatment is proof-of-concept inhibitor of LIMD1 deficient lung cancers.* A)** Tumour volume of subcutaneous isogenic A549 xenografts implanted into the flank of NOD/SCID mice. Mice were treated twice on indicated days with vehicle of PF-477736 (7.5 mg/kg per dose) (n = 10 per group, two-way ANOVA). **B)** Tumour volume of xenografts at day 29 (n = 10 per group, two-way ANOVA). **C-D)** Example staining of Ki67 and cleaved caspase-3 in xenografts. Arrows in panel D indicate cleaved caspase-3 positive cells.
