# Supplementary figures and images for: Targeted therapy for LIMD1-deficient non-small cell lung cancer subtypes
Source: Cell Death Dis. 2021 Nov 11;12(11):1075. doi: 10.1038/s41419-021-04355-7 (PMC8586256; doi:10.1038/s41419-021-04355-7)

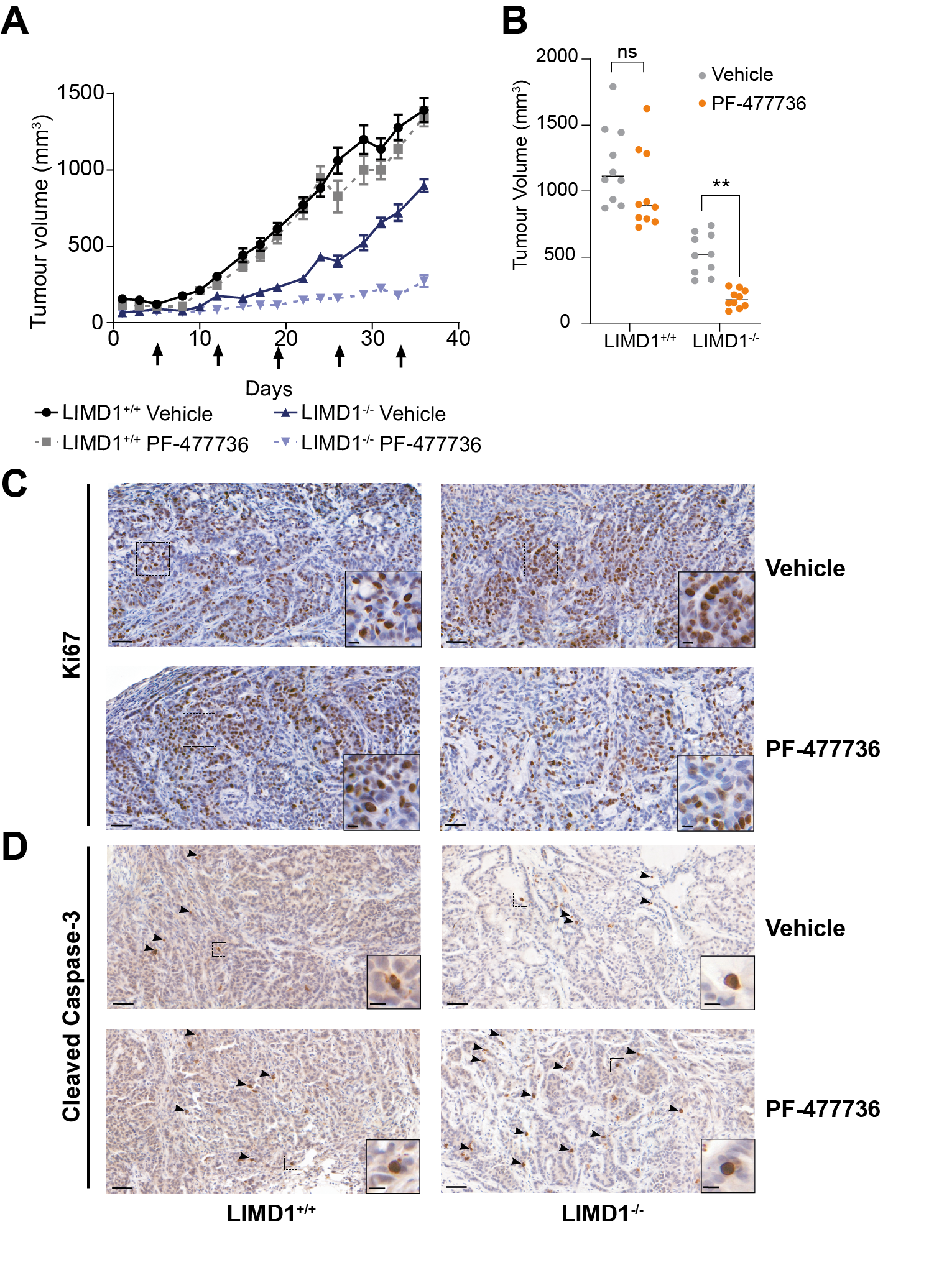

Supplement: Supplementary file 2 — Supplemental Fig. 4. [file 41419_2021_4355_MOESM2_ESM.png]

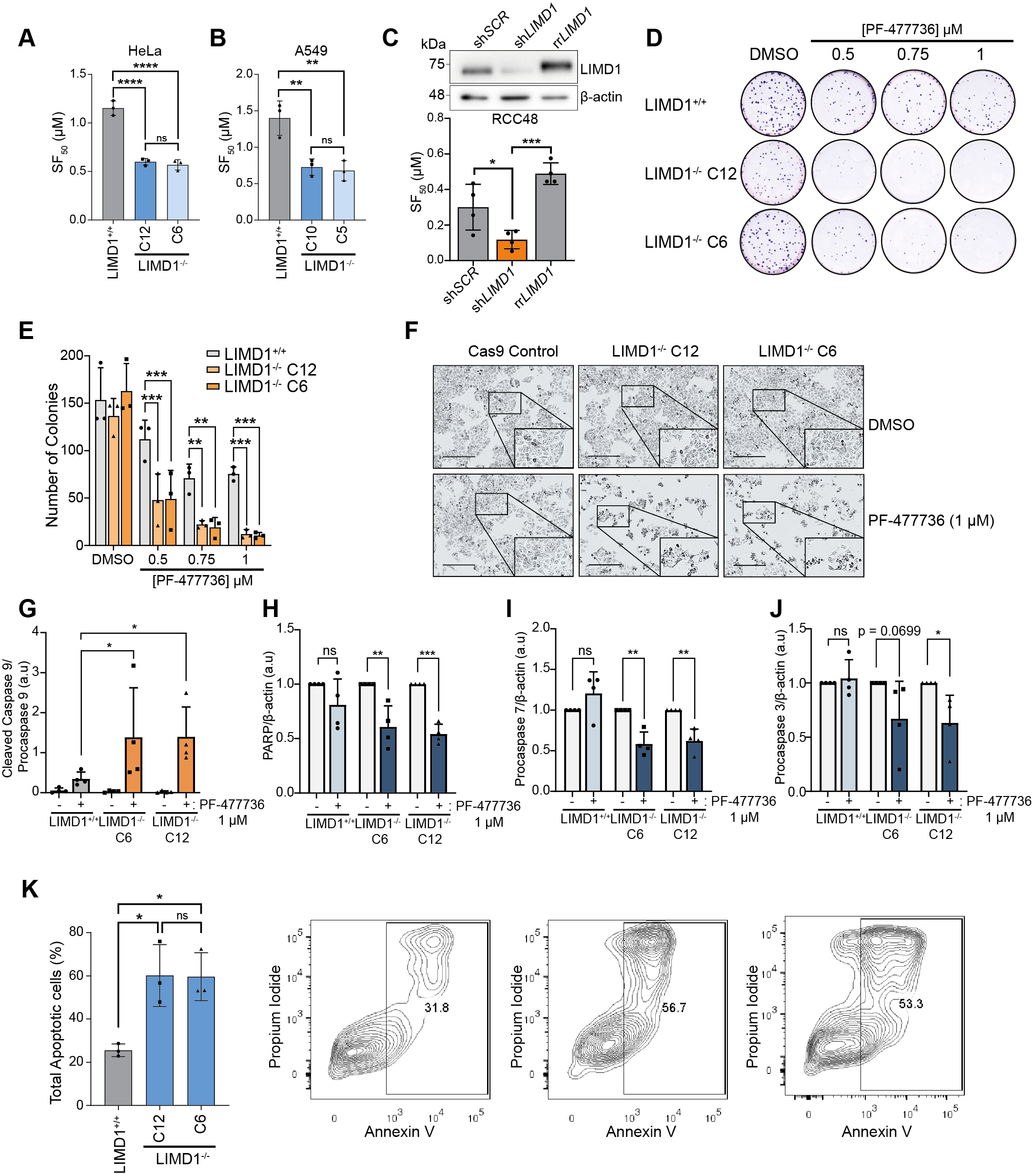

Supplement: Supplementary file 3 — Supplemental Fig. 1. [file 41419_2021_4355_MOESM3_ESM.png]

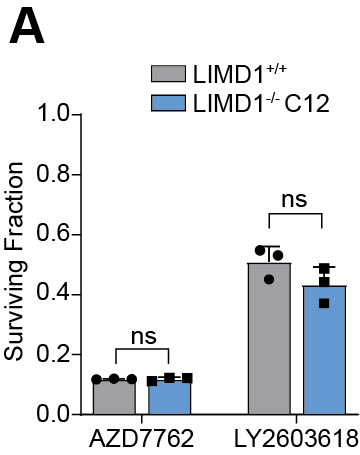

Supplement: Supplementary file 4 — Supplemental Fig. 2. [file 41419_2021_4355_MOESM4_ESM.png]

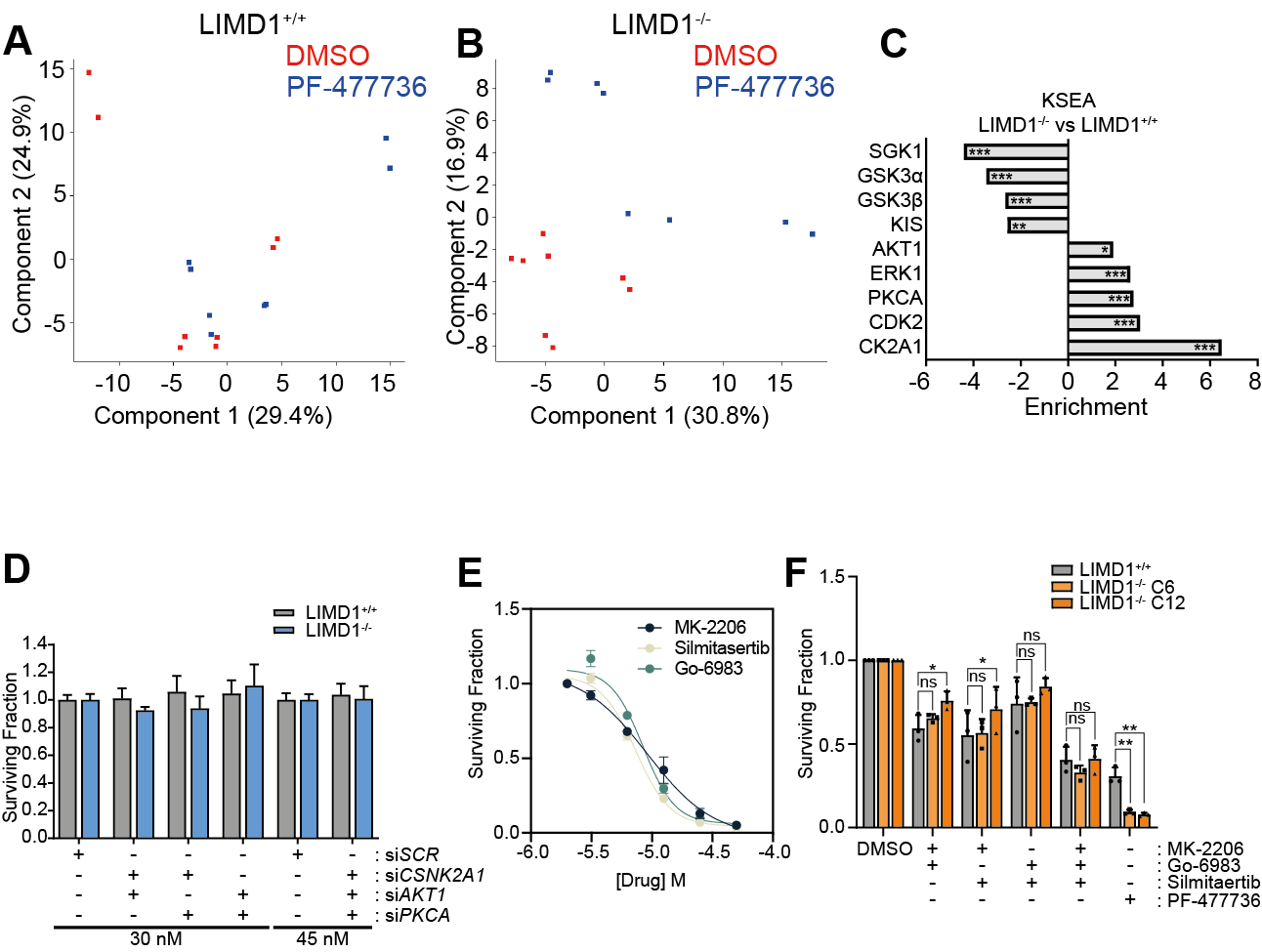

Supplement: Supplementary file 5 — Supplemental Fig. 3. [file 41419_2021_4355_MOESM5_ESM.png]
